# Clinical Anatomy of the Frenulum of the Oral Vestibule

**DOI:** 10.7759/cureus.1410

**Published:** 2017-06-29

**Authors:** Joe Iwanaga, Naoshi Takeuchi, Rod J Oskouian, R. Shane Tubbs

**Affiliations:** 1 Seattle Science Foundation; 2 Department of Periodontology, Kagoshima University Graduate School of Medical and Dental Sciences; 3 Neurosurgery, Complex Spine, Swedish Neuroscience Institute; 4 Neurosurgery, Seattle Science Foundation

**Keywords:** mimetic muscle, labial frenulum, buccal frenulum, anatomy, cadaver

## Abstract

Introduction

The frenula of the oral vestibule include the labial and buccal frenula. Abnormal labial and buccal frenula can affect facial esthetics and oral cavity function by retracting the gingival margin, creating a median diastema, and limiting lip movement. Because of the lack of information on these structures, we aimed to clarify their anatomy.

Methods

A total of 34 sides from 17 fresh frozen cadaveric Caucasian heads were used in the present study. The specimens were derived from 11 male and 6 female adult cadavers. The relationships between the frenulum of the mucosa and the tissue underneath the mucosa was observed.

Results

The buccal frenulum was formed by the border of mimetic muscles and connective tissues. Comparitively, the labial frenulum was only formed by taut connective tissue.

Conclusion

We found that the buccal and labial frenula have different compositions. This finding may have relevance both in oral surgery and in various cosmetic procedures near the oral vestibule.

## Introduction

The frenula in the oral vestibule consist of the labial and buccal frenula. The labial frenulum is located in the middle of the oral vestibule. The upper and lower labial frenula are commonly attached below the upper and lower alveolar crests, respectively [1]. Histological observation of the labial frenulum has demonstrated the existence of the epithelium and muscle fibers [2]. The connective tissue between the inferior portion of the incisivus labii superioris (ILS) muscle corresponded to the inferior labial frenulum and the extent of the inferior part of the ILS corresponded to the folds of the upper alveolar mucosa. The lower labial and buccal frenula occasionally cause the recession of the gingiva and can interfere with dentures. The connective tissue between the inferior portion of the ILS muscle corresponded to the inferior labial frenulum and the extent of the inferior part of the ILS corresponded to the folds of the upper alveolar mucosa. The lower labial and buccal frenula occasionally cause recession of the gingiva and can interfere with dentures. The connective tissue between the upper portion of the mentalis muscle (MT) corresponded to the inferior labial frenulum, and the extent of the upper portion of the MT corresponded to the folds of the lower alveolar mucosa [3]. Clinically, an abnormal labial or buccal frenulum can retract the gingival margin, create a median diastema, limit the movement of the lip, and affect esthetics [4]. Frenulectomy is required to treat these anomalies and several modified procedures have been developed [4-5]. However, there have been insufficient anatomical studies of the frenula of the oral vestibule, especially the buccal frenulum. Therefore, we aimed to clarify the anatomical structure of the frenula in the oral vestibule for a better understanding of surgical procedures and general dental practice.

## Materials and methods

A total of 34 sides from 17 fresh frozen adult cadaveric Caucasian specimens were used in the present study. These were derived from 11 males and 6 females and the age of the cadavers at death ranged from 67 to 99 years old (mean age: 79.8 ± 9.3 years). Before dissection, the upper and lower buccal frenula were observed and the positions of the frenulum were recorded. The initial mucosal incision was made horizontally into the microgingival junction, running from the right to the left molar region. Next, the mucosa was elevated from the site of the horizontal incision toward the mucodermal junction. The relationship between the frenulum of the mucosa and the tissue underneath the mucosa was observed. All dissections were carried out under a surgical microscope (OPMI CS NC31, Carl Zeiss, Oberkochen, Germany). The present cadaveric study was performed in accordance with the requirements of the Declaration of Helsinki (64th WMA General Assembly, Fortaleza, Brazil, October 2013).

## Results

**Figure 1:**
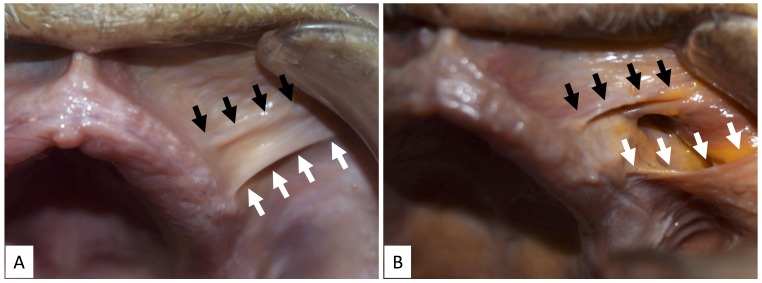
Buccal frenulum of the left maxilla A: Before the removal of the mucosa B: After the removal of the mucosa Black arrows: lateral border of the incisivus labii superioris (ILS), white arrows: anterior border of the buccinator

The buccal frenulum of the maxilla, formed around the canine region, corresponded to the lateral border of the lower portion of the ILS. The buccal frenulum of the maxilla, formed in the premolar region, corresponded to the anterior border of the buccinator (Figure 1).

**Figure 2:**
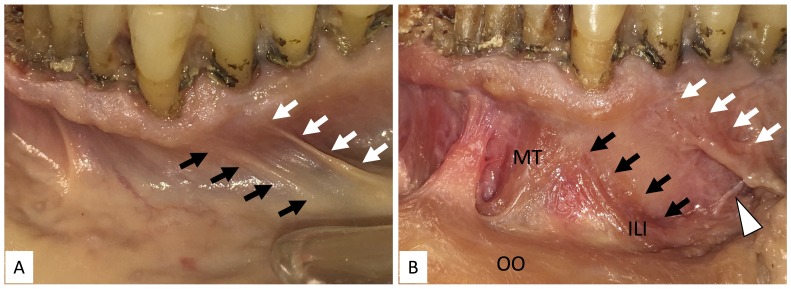
Buccal frenulum of the left mandible A: Before the removal of the mucosa B: After the removal of the mucosa (the MT and connective tissue underneath the lower labial frenulum have been separated) Black arrows: lateral border of the upper part of the MT and ILI, white arrows: anterior border of the buccinator, arrowhead: mental foramen, ILI: incisivus labii inferioris muscle, MT: mentalis, OO: orbicularis oris

The buccinator fibers that attached to the maxilla went deep to the mucosa that attached to the mucogingival junction. The buccal frenulum of the mandible, formed around the canine region, corresponded to the lateral border of the upper portion of the MT and incisivus labii inferioris (ILI) (Figure 2).

**Figure 3:**
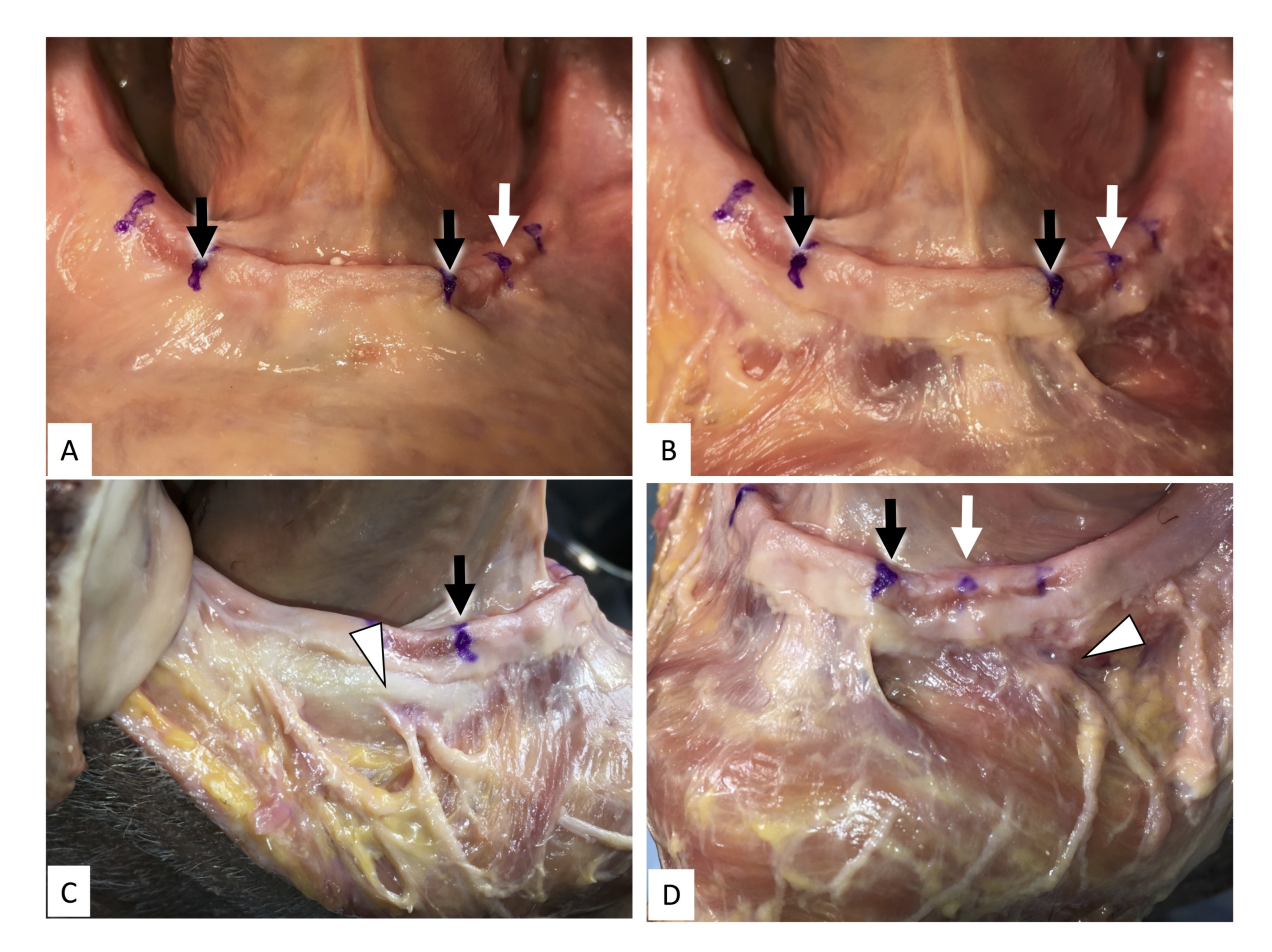
Second buccal frenulum of the mandible A: Before the removal of the mucosa B: After the removal of the mucosa C: Lateral view (right) of the buccal frenulum of the mandible D: Lateral view (left) of the buccal frenulum of the mandible; note that the white arrow indicates the second buccal frenulum Black arrows: lateral border of the upper part of the mentalis, white arrows: lateral border of the ILI, arrowhead: mental foramen

When the bony attachment of the upper portion of the MT and ILI had a gap, a second buccal frenulum was observed (Figure 3).

The buccal frenulum of the mandible, formed in the premolar region, corresponded to the anterior border of the buccinator muscle. The buccinator muscle fibers that attached to the mandible also attached to the mucogingival junction. In 16 cases, the upper labial frenulum was observed more clearly than the lower labial frenulum. In one case, the lower labial frenulum was very clear and similar to the upper frenulum, which has the higher attachment of the taut connective tissue underneath the frenulum and near the orbicularis oris. The connective tissues found underneath the upper and lower labial frenula were composed of different structures; the former was a taut and thin connective tissue, the latter was taut but thick and flat and occupied the space surrounded by the left and right mentalis and binding these muscles.

## Discussion

Anatomical and histological studies of the frenulum have been reported [6-9]. These studies, which included histological findings, are currently evidence for surgical procedures treating a high-positioned frenulum [10-11]. However, it seems that in the past, frenulectomy was carried out without reliable anatomical data or research into exactly what the frenulum is. Anatomy textbooks only briefly describe the frenulum [1,12]. One of the reasons for the lack of anatomical data is that most anatomical studies use embalmed cadavers in which the details of the frenulum are very difficult to discern. The results of our study clearly showed that the border of the muscles and associated connective tissues formed the buccal frenulum. The muscle attached to the mucosa so that it did not form the frenulum but, instead, the taut connective tissue formed the labial frenulum. The bony attachment of these taut connective tissues can be affected by the attachment of the MT onto the mandible and the ILS onto the maxilla. The origin of these frenula was completely different. Therefore, these two frenula should probably be treated in different ways.

Although the anatomy of the labial frenulum has been described, to our knowledge, there have been scant studies that have described the structures deep to the buccal frenulum. Traditionally, in terms of periodontology, the buccal frenulum is considered as possibly one of the causes of gingival recession. According to Toker and Ozdemir [13], a high frenulum is correlated to gingival recession, and gingival recession of the mandible was significantly higher than that of the maxilla. However, our results showed that the buccal frenulum was just the outline of the muscle and in this light, it is difficult to believe that the frenulum could be the cause of gingival recession. There might be physiological reasons for gingival recession other than anatomical ones. The mobility and extension direction of the mucosa on either side of the buccal frenulum can be different because these mucosae are supported by different muscles and connective tissues. The buccal and labial frenula are also important for complete denture impression [14-15]. It has been considered that the levator anguli oris (caninus muscle) lies beneath the buccal frenulum for the maxilla, and the depressor anguli oris sits beneath the mandible [15-16]. However, the results of our intraoral dissections of fresh cadavers were different. Also, a difference between these structures deep to the upper and lower labial frenula might result in a difference in visualizing the labial frenula. The flat shape of the connective tissue might be the reason why the lower labial frenulum is not identified in many cases when the lower lip is relaxed, and the thin connective tissue of the upper labial frenulum might be the reason why the upper labial frenulum can be identified in many cases even when the upper lip is relaxed. In the present study, although the insertion of the taut connective tissue in the midline was not measured in detail, the lower insertion of the taut connective tissue into the orbicularis oris (OO) of the upper lip and the higher insertion of the taut connective tissue into the OO of the lower lip possibly make the upper and lower labial frenula more clear. Although we did not measure the thickness of the ILS, mentalis, incisivus labii inferiorosis (ILI), and buccinator muscles in this study, this might affect the visibility of the buccal frenulum. 

## Conclusions

We dissected the frenula of the oral vestibule in 34 sides from 17 fresh frozen cadaveric Caucasian heads and found that the borderline between the muscles and connective tissue forming the buccal frenulum is completely different from that forming the labial frenulum. The buccal frenulum of the maxilla, formed around the canine region, corresponded to the lateral border of the lower portion of the ILS. The buccal frenulum of the maxilla, formed in the premolar region, corresponded to the anterior border of the buccinator. The buccal frenulum of the mandible, formed around the canine region, corresponded to the lateral border of the upper portion of the MT and ILI. The buccal frenulum of the mandible, formed in the premolar region, corresponded to the anterior border of the buccinator muscle.

## References

[1] Standring S (2015). Gray's anatomy: the anatomical basis of clinical practice. https://books.google.com/books?hl=en&lr=&id=b7FVCgAAQBAJ&oi=fnd&pg=PP1&dq=Gray%27s+Anatomy:+The+Anatomical+Basis+of+Clinical+Practice,+41e&ots=4Nn-HYkHnw&sig=8L9xssCP9Drz9_Al05B_o1exbK4#v=onepage&q=Gray's%20Anatomy%3A%20The%20Anatomical%20Basis%20of%20Clinical%20Practice%2C%2041e&f=false.

[2] Gartner LP, Schein D (1991). The superior labial frenum: a histologic observation. Quintessence Int.

[3] Hur MS, Kim HJ, Choi BY (2013). Morphology of the mentalis muscle and its relationship with the orbicularis oris and incisivus labii inferioris muscles. J Craniofac Surg.

[4] Bagga S, Bhat KM, Bhat GS (2006). Esthetic management of the upper labial frenum: a novel frenectomy technique. Quintessence Int.

[5] Koora K, Muthu MS, Rathna PV (2007). Spontaneous closure of midline diastema following frenectomy. J Indian Soc Pedod Prev Dent.

[6] Gottsegen R (1954). Frenum position and vestibule depth in relation to gingival health. Oral Surg Oral Med Oral Pathol.

[7] Sewerin I (1969). Frenulum labii superioris. Anatomical variations and abnormalities (Article in Danish). Tandlaegebladet.

[8] Sewerin I (1971). Prevalence of variations and anomalies of the upper labial frenum. Acta Odontol Scand.

[9] Henry SW, Levin MP, Tsaknis PJ (1976). Histologic features of the superior labial frenum. J Periodontol.

[10] Edwards JG (1977). The diastema, the frenum, the frenectomy: a clinical study. American journal of orthodontics. Am J Orthod.

[11] Devishree Devishree, Gujjari SK, Shubhashini PV (2012). Frenectomy: a review with the reports of surgical techniques. JCDR.

[12] Netter FH (2012). Atlas of human anatomy. https://books.google.com/books?hl=en&lr=&id=0yMPDQAAQBAJ&oi=fnd&pg=PP1&dq=Atlas+of+human+anatomy+2012&ots=8tiCYDh-ip&sig=l_qafEEkEP20aETc418C0MC8Nao#v=onepage&q=Atlas%20of%20human%20anatomy%202012&f=false.

[13] Toker H, Ozdemir H (2009). Gingival recession: epidemiology and risk indicators in a university dental hospital in Turkey. Int J Dent Hyg.

[14] Boucher CO (1944). Complete denture impressions based upon the anatomy of the mouth. JADA.

[15] Boucher CO (1951). A critical analysis of mid-century impression techniques for full dentures. J Prosthet Dent.

[16] Sarandha DL, Hussain Z, Uthkarsh Uthkarsh (2008). Textbook of complete denture prosthodontics. https://www.abebooks.com/9788184480894/Textbook-Complete-Denture-Prosthodontics-Sarandha-818448089X/plp.

